# Long-Term Efficacy of the Combination of Active Vision Therapy and Occlusion in Children with Strabismic and Anisometropic Amblyopia

**DOI:** 10.3390/children9071012

**Published:** 2022-07-07

**Authors:** Myriam Milla, Ainhoa Molina-Martín, David P. Piñero

**Affiliations:** 1 Oftalvist, 03013 Alicante, Spain; mmilla@oftalvist.es; 2 Department of Optics, Pharmacology and Anatomy, University of Alicante, 03690 Alicante, Spain; ainhoa.molina@ua.es

**Keywords:** amblyopia, anisometropia, occlusion therapy, patching, strabismus, stereopsis, vision therapy

## Abstract

This retrospective study was conducted to evaluate the efficacy of the combined treatment of occlusion and active vision therapy in a total of 27 amblyopic children, including 14 strabismic and 13 anisometropic cases. For such purpose, changes in distance and near visual acuity as well as in the binocular function was evaluated during a two-year follow-up. In both amblyopia groups, significant improvements were found in distance and near visual acuity in the non-dominant eye (*p* < 0.001). In the strabismic amblyopia group, the percentage of patients with binocular function score (BF) > 3.3 decreased significantly from a baseline value of 64.3% to a two-year follow-up value of 7.1% (*p* < 0.001). In the anisometropic amblyopia group, this percentage also decreased significantly from a baseline value of 15.4% to a two-year follow-up value of 0.0% (*p* < 0.001). No recurrences were observed in the anisometropic amblyopia group, whereas recurrence occurred in two cases of the strabismic amblyopia group after finishing the vision rehabilitation process. In conclusion, the combined approach of the treatment evaluated is efficacious for providing an improvement in visual acuity and binocular function in both anisometropic and strabismic amblyopia, which was maintained over time.

## 1. Introduction

Amblyopia is a neurological disorder that affects the development of the visual system in early life [1,2,3]. Several studies have reported and listed the differences that are present between strabismic and anisometropic amblyopia [4,5], this differential behavior being an important factor when considering the protocol for amblyopia rehabilitation. The visual acuity reduced in one or both eyes is the visible part of a number of common deficits such as decreased contrast sensitivity (for high frequencies in strabismic amblyopia and limitation across the frequency range in anisometropic amblyopia), slow and uneven accommodative response, a crowding effect (stronger in strabismic), and reduced or absent stereopsis [4,5,6,7]. However, strabismic amblyopia encompasses other problems that further hinder the improvement of visual function. Reduced cortical control of movement due to strabismus [8], combined with amblyopia, leads to reversals in improvement even when treatment is considered completed.

The active methods of treatment were born to complement passive methods such as occlusion or atropine in the dominant eye [9]. Active visual rehabilitation focuses on improving amblyopia by capturing the patient’s attention during the treatment period, as well as by activating the connectivity of certain cell types at the cortical level [9,10]. The new approach to active visual rehabilitation methods for the treatment of amblyopia has led to the development of new protocols based on the following techniques: perceptual learning, dichoptic training, binocular vision therapy and virtual reality [11,12,13,14]. In anisometropic amblyopia, according to several studies, the best option seems to be the combination of active vision therapy and occlusion, addressing not only the improvement of visual acuity and stereopsis, but also the rest of the visual skills reduced by this type of amblyopia [5]. In strabismic amblyopia, active visual therapy under binocular conditions should be performed after ensuring that bifoveal fusion is present, this being a limiting factor for treatment with dichoptic training [4]. Furthermore, in most studies on vision therapy in amblyopia, only a minimal part of the patients included are diagnosed with strabismic amblyopia and, therefore, it is difficult to draw specific conclusions for this type of amblyopia and the success of active vision therapy.

The main objective of this study was to evaluate the efficacy of the combined treatment of occlusion and active vision therapy in both strabismic and anisometropic amblyopia by evaluating the retrospective data collected in a pediatric ophthalmology unit of a private hospital.

## 2. Materials and Methods

### 2.1. Patients

This retrospective study analyzed data from all patients evaluated and treated from February 2017 to December 2021 in Oftalvist Clinic (Alicante, Spain). This study was conducted in accordance with the tenets of the Declaration of Helsinki, and with the approval of the institutional ethics committee of University of Alicante (UA-2018-03-03). Inclusion criteria were children with strabismic and anisometropic amblyopia following a complete program of visual rehabilitation in our institution. Exclusion criteria were a history of other ophthalmological or systemic diseases, eccentric fixation, amblyopia fully recovered with optical correction, previous history of amblyopia therapy, congenital cataracts, and nystagmus. Clinically, amblyopia was defined as the presence of two conditions: one or both eyes having a visual acuity of 6/12 or worse, and one or more lines of difference in logMAR visual acuity between the eyes in unilateral amblyopia [4,15]. In this study, anisometropic amblyopia was defined as an amblyopia associated to a significant difference in the magnitude of refractive error between both eyes (difference ≥ 1.00 D in the equivalent sphere between eyes and/or difference ≥ 1.50 D in astigmatism between corresponding meridians between eyes), and without the presence of strabismus [5]. Diagnosis of strabismic amblyopia was considered as the combination of amblyopia with constant strabismus in addition to refractive error [4].

### 2.2. Examination Protocol

All subjects completed an ophthalmologic and optometric examination to confirm the diagnosis of amblyopia, including autorefraction, tonometry, measurement of distance and near LogMAR visual acuity, manifest and cycloplegic refraction (cyclopentolate 1%), fundus exam, ocular motility test, cover test (exo- and eso-deviation were recorded by − and +, respectively), objective evaluation of fixation behavior (microperimetry, MAIA system, Centervue, Padova, Italy), cover test, and stereopsis. Similarly, a complete baseline examination before vision therapy was also performed. All patients began with spectacle correction and started an occlusion therapy according to PEDIG guidelines [16] after a period of refractive adaptation of four to six weeks. Data from the follow-up visits were registered and analyzed at: baseline, 15 days, one month, two months and three months during monocular and binocular training. The evolution of the possible improvement in binocular vision was measured with the TNO test, giving a range of values according to the state of stereoscopic vision. The BF (binocular function score) was calculated with value 5 representing suppression, value 4 simultaneous vision or flat fusion. and from 1.6 to 3.3 (log 40 arc sec − log 2000 arc sec) the presence of stereopsis [17].

Each patient performed monocular training in the amblyopic eye during three months at home and at office. Then, by restoring part of visual acuity in the non-dominant eye, all patients combined vision therapy with perceptual learning training.

### 2.3. Active Vision Therapy

**Figure 1:**
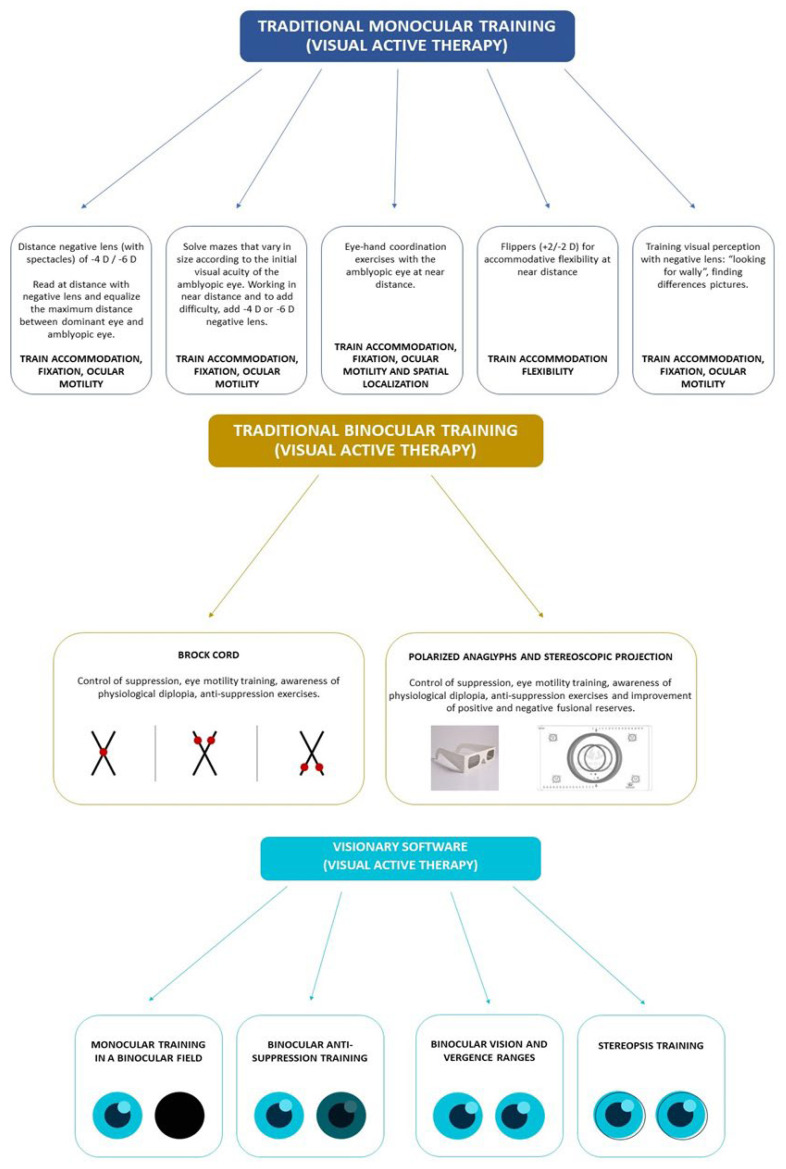
Diagram showing the type of vision therapy performed in the current study.

The training procedure was performed both in the home and the office settings, but always under the professional supervision of the same optometrist (M.M.). All patients underwent a program of personalized visual exercises according to age and cognitive level. Figure 1 shows a diagram explaining the type of procedures used in the vision therapy program. The rehabilitative protocol followed in our center in amblyopia began with an intensive training of the monocular visual function, including the accommodative response, ocular motility and spatial localization, all of which are affected in amblyopia [17]. Occlusion was remained in this initial stage of treatment as an additional method of monocular treatment (passive), following the PEDIG guidelines [16]. Once the patient reached a visual acuity of 20/30 in the amblyopic eye, occlusion was stopped and binocular training was initiated, as the cortical integration of images can be facilitated without a very significant worsening of the non-amblyopic eye. Occlusion was not prescribed if the visual acuity in the amblyopic eye was better than 20/30. In strabismic amblyopia cases, the correction of the deviation with surgery or prisms (if normal retinal correspondence was presence and the deviation was 12 prism diopters or below) was required before initiating the binocular training [18]. A weekly or biweekly training session at the office of around 30–45 min was performed with an additional daily home exercise routine of approximately 20–30 min. This part of the treatment was successfully performed by all patients. As traditional exercises, such as flippers or printed sheets of tests, were used, it was impossible to check the exact compliance of the treatment. A successful outcome was achieved when obtaining a 1% frequency of tropia provided that diplopia is noticed at these times and up to five prism diopters was required to be worn in spectacles (Flom’s criteria) [19].

Once finished the monocular training, binocular training was initiated with traditional tools, such as a Brock cord or anaglyphs, following the same program of visits and home exercises of monocular training. This binocular stimulation was performed until achieving a gross stereopsis of 480 arc sec, with stimulation of fine stereopsis afterwards with 20–30 min of home exercises using the specialized software Visionary (Visionarytool, Gijón, Spain). The use of this software has been demonstrated to be effective for improving the level of stereoacuity in amblyopes [20]. If fine stereopsis was present after monocular training, this specialized software was used directly. The treatment stopped once achieving a stereopsis level of 120 arc sec or one year after performing active training without improving from gross stereopsis or not achieving it. All patients use the Visionary software as a maintenance method (20 min of training) for six months after finishing the rehabilitation program. The optical correction was maintained during the follow-up if it was needed to maintain good visual acuity and the alignment.

Recurrence was defined as a 0.2 or more logMAR loss of visual acuity according to Walsh et al. [21]. No recurrences were observed in the anisometropic amblyopia group, whereas recurrence occurred in two cases of the strabismic amblyopia group after finishing the vision rehabilitation process. In these two cases, the binocular stimulation was initiated again, fully recovering the visual loss at the end of the follow-up.

### 2.4. Statistical Analysis

Statistical analysis of the results was done using the SPSS program v.19.0.0 for Windows (SPSS Inc., Chicago, IL, USA). Analysis of normality by Kolmogorov-Smirnov test revealed that most of the parameters did not follow a normal distribution, so, accordingly non-parametric tests were applied. The Friedman test was used to assess the statistical significance of differences in the anisometropic and strabismic groups between consecutive visits, with a post-hoc comparison by pairs using the Wilcoxon test adjusted with the Bonferroni correction. Concerning percentage, differences between baseline and last visit were evaluated with the McNemar test. Differences were considered to be statistically significant when the associated *p*-value was <0.05. The results reported in were those obtained once the period of refractive adaptation was finished and therefore changes were due to the combination of active (training) and passive therapy (occlusion) of amblyopia.

As the sample of patients recruited was small, the statistical power associated to each change that was found to be statistically significant was calculated a posteriori using the software PS Power and Sample Size Calculations Version 3.0 (Vanderbilt University, Nashville, TN, USA). This software allows for the performing of a calculation of the statistical power associated with different types of statistical tests using the method described by Dupont and Plummer [22]. The statistical power was calculated for changes in distance BCVA and BF.

## 3. Results

A total of 27 patients were analyzed, 14 were strabismic amblyopes and 13 were anisometropic amblyopes. The mean age in the strabismic amblyopia group was 11.0 ± 2.7 years (range, 7–16 years), whereas in the anisometropic amblyopia group it was 11.0 ± 2.6 years (range, 8–18 years).

### 3.1. Sample Size Calculations

Table 1 shows the statistical power calculations for the anisometropic and strabismic amblyopia groups associated with the analysis of changes at the end of follow-up compared to the baseline conditions in distance BCVA and BF. As shown, the statistical power associated to distance BCVA and BF changes in both strabismic and anisometropic groups were over 95.0%.

### 3.2. Strabismic Amblyopia

Table 2 reports the data obtained according to the non-amblyopic and amblyopic eye and according to visits to the office (initial, 15 days, 30 days, 60 days, 90 days, one year and years years). In addition, the evolution of binocular vision results in terms of cover test and BF was provided. The analysis of BCVA and NVA (best corrected visual acuity and near vision visual acuity) outcomes revealed the presence of no statistically significant changes (*p* = 0.73 and *p* = 0.11, respectively) in the non-amblyopic eyes as the treatment progressed, even at one and two years of follow-up. However, in the analysis of amblyopic eyes, significant improvements were observed for both BCVA and NVA (*p* < 0.01). In the comparison by pairs, statistically significant differences were found in BCVA between the first visit and the rest of visits after one month of combined treatment (*p* = 0.02). In the NVA analysis, according to the analysis by pairs, statistically significant differences were only found between the results obtained at the visit after 90 days of combined treatment and after one year of follow-up with respect to the initial visit (*p* = 0.02 and *p* = 0.04, respectively). In terms of cycloplegic and subjective refraction, no statistically significant changes were observed between visits in both the non-amblyopic and amblyopic eyes (*p* > 0.05).

**Figure 2:**
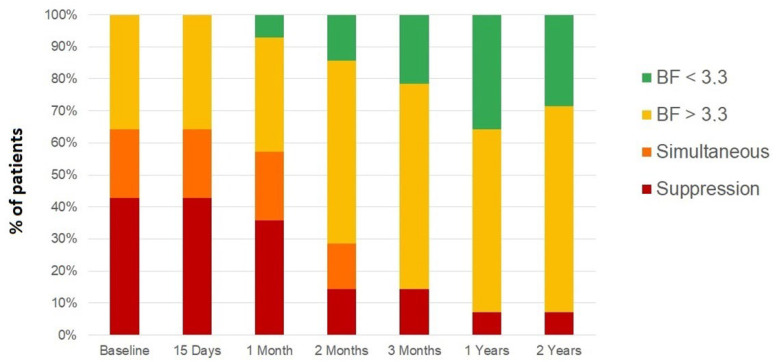
Binocular function (BF) score evolution of strabismic amblyopia group from the previous visit to 15 days, 30 days, 60 days and 90 days of treatment, as well as at one and two years after the completion of treatment.

The evolution of binocular vision was recorded by improvements in the BF. At the first visit, of the 14 patients with strabismic amblyopia, nine of them had suppression or simultaneous vision, with only five showing some degree of gross stereopsis without reaching bifoveal fixation. After three months of combined treatment (occlusion and active visual therapy), only two patients maintained the state of binocular suppression, nine patients achieved some degree of gross binocular vision, and only two achieved random stereopsis. After one and two years of follow-up, the results obtained after treatment were maintained, with only one patient with suppression and 13 patients with some degree of gross binocular vision or random stereopsis (Figure 2). The percentage of patients with BF > 3.3 decreased significantly from a baseline value of 64.3% to a two-year follow-up value of 7.1% (*p* < 0.001).

Concerning the type of deviation, only one case of exotropia was included while the rest of the cases were esotropias. The case of exotropia (17 prism diopters at distance and 25 prism diopters at near) showed an improvement of BF from 2.60 at baseline to 1.80 at the end of the follow-up, this being the case with the highest post-therapy BF of the sample.

### 3.3. Anisometropic Amblyopia

Table 3 shows the results obtained from the analysis in the group of anisometropic amblyopes. In the non-amblyopic eyes, no statistically significant changes were found in BCVA between visits (*p* = 0.20). In contrast, significant differences were found in NVA (*p* = 0.01). However, after the Bonferroni correction of paired comparisons, no statistically significant differences were found between pairs of visits (*p* > 0.05). There was only a trend in non-amblyopic eyes to a slight improvement in NVA after treatment compared to baseline. In amblyopic eyes, statistically significant changes were observed in both BCVA and NVA results (*p* < 0.05). According to the analysis by pairs in BCVA, differences between visits were found to be significant when comparing the 15 and 30-day visits of combined treatment and the one-year and two-year follow-up with respect to the initial (*p* = 0.02) in all cases. Likewise, differences were statistically significant between the 15-day visit and the two-year follow-up (*p* = 0.02) as well as between the first month’s visit with respect to two-year follow-up (*p* = 0.04). In the NVA analysis by pairs in non-amblyopic eyes, no significant differences between visits were found. Finally, subjective and cycloplegic refraction did not show statistically significant changes during the follow-up (*p* > 0.05).

**Figure 3:**
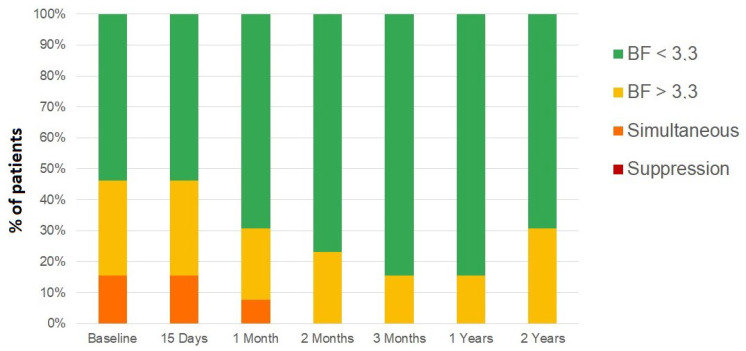
Binocular function (BF) score evolution of anisometropic amblyopia group from the previous visit to 15 days, 30 days, 60 days and 90 days of treatment, as well as at one and two years after completion of treatment.

The initial status of the 13 patients with anisometropic amblyopia was as follows: two patients had flat fusion or simultaneous vision, four had gross stereopsis, and seven had random fine stereopsis. After the evolution of combined occlusion and vision therapy, 11 patients achieved fine stereopsis and only two coarse stereopsis. After the evolution and follow-up of one and two years after the end of the treatment, the results of the BF value were maintained (Figure 3), with the great majority of patients achieving fine stereoacuity with bifoveal fixation and three patients still having gross stereopsis. The percentage of patients with BF > 3.3 decreased significantly from a baseline value of 15.4% to a two-year follow-up value of 0.0% (*p* < 0.001).

## 4. Discussion

In the current series, a combined treatment of occlusion and active vision therapy has been shown to improve the visual function in both anisometropic and strabismic amblyopia, demonstrating that this approach may be a good and integral option to provide a visual rehabilitation in amblyopia. Although some studies have demonstrated the ability of patching of providing some improvements in stereopsis and contrast sensitivity [23,24,25], there are other options in terms of active visual training that have also been shown to provide an effective rehabilitation of the binocular function of amblyopia, including perceptual learning training, accommodative and binocular function stimulation [4,5]. However, this does not mean that this active vision therapy is a substitute for patching [26]. Indeed, previous experiences have shown the benefit of the synergistic combination of patching and vision therapy, leading to satisfactory results, even in those cases treated unsuccessfully only with patching [18,27]. Likewise, the difference between the course of the treatment in strabismic and anisometropic amblyopia has been also demonstrated, with a slower recovery and the achievement of less degree of binocular vision in strabismic amblyopia.

This was not a comparative study to confirm if vision therapy is better than patching, as in all patients both treatments were used in combination. Different previous investigations have already demonstrated the efficacy and indications of patching or vision therapy to treat amblyopia [4,5,14,16,23,24,25,28,29,30,31,32,33,34,35,36,37,38,39,40,41]. However, the results of the combination of treatments in amblyopia are still scarce [18,27]. In the current study, a retrospective analysis of the long-term results of our clinical practice is provided by clearly differentiating the results of anisometropic and strabismic amblyopic patients and filtering the cases included, ensuring that none of them had previous ocular surgeries, occlusion or vision therapy treatments. From our perspective, the report of the results of amblyopia treatments in the long term in order to confirm the recurrence rate and potential predicting factors for this situation is especially interesting. The results from our series must be confirmed in future prospective comparative studies, including randomized clinical trials confirming whether the combined treatment option provides a significant benefit over the prescription of a single treatment, occlusion or vision therapy.

The group of strabismic amblyopia in the current series showed improvements in distance and near visual acuity as well as in the binocular function scoring. The recovery of visual acuity occurred mainly during the first two to three months, whereas the improvement of the binocular function began at three months and was therefore a later change. This could be explained due to the sequence of treatment in these cases, including an initial active and passive monocular stimulation, and the initiation of a binocular phase once the monocular recovery was significant (around 0.2 logMAR). This phase could be initiated then after two to three months or more of treatment. Binocular vision training should be considered as the final part of the treatment in strabismic amblyopia and should always confirm if a bifoveal fixation was possible (no sensorial adaptations present) [4]. Molina-Martin et al. [18] evaluated the results of the combination of passive and active treatment for the management of amblyopia in esotropic subjects with accommodative component. These authors refracted all subjects under cycloplegia and treated them with occlusion (passive therapy), as in the current series. After a period of adaptation, subjects not achieving orthotropia with the optical correction performed an active vision therapy (full-time prismatic correction and subsequent fusional vergence therapy), the performance of surgery in larger angles (>12 prism diopters) being necessary [18]. A similar protocol was followed in the current series, but it should be considered that exotropias were also included in which the optical correction has a minimal effect on ocular alignment. With the protocol mentioned, Molina-Martin et al. [18] found that all subjects acquired stereoacuity equal or better than 800’’, besides a significant visual acuity improvement. In our series, there was a small portion of patients (7.1%) remaining with suppression despite experiencing a visual acuity improvement. As mentioned, our series included large angle constant exotropia cases as well as non-accommodative esotropias, and some of them may have a worse prognosis. It should be considered that the significant changes detected in the ocular deviation in our cases of strabismic amblyopia over time was mainly due to the reduction or elimination of some tropias with surgery.

The group of anisometropic amblyopia responded very favorably to both active vision therapy and binocular control in the early phases of the treatment, with the achievement of stereopsis by all patients. This suggests that the prognosis of an efficacious visual rehabilitation with the combined treatment described is better in anisometropic amblyopia. This is consistent with previous research showing significant differences in the neural mechanism of both types of amblyopia, with significant differences in interhemispheric functional connectivity [42,43,44]. Indeed, more limited outcomes of dichoptic or binocular therapies have been reported in those samples of amblyopes including a relevant proportion of strabismic amblyopes [45]. Although more studies are needed, it is important to consider this when explaining to patients or parents the prognosis of the treatment of their amblyopia or the amblyopia of their children. As shown, the achievement or improvement of stereopsis in anisometropic amblyopia seems to be a common finding among studies [46], but depending on the specific features of each case, this may be not be possible in strabismic amblyopia. A careful analysis of each case of strabismic amblyopia must be performed in the clinical setting to select the most appropriate patient management, combining, in most of cases, passive and active therapies.

In the last years, a great variety of studies have shown the outcomes of active vision therapy under dichoptic environments in most of the cases in amblyopia, specially associated to anisometropia [28,29,30,31,32,33,34,35,36,37,38,39,40]. However, few studies have investigated the potential of combining both patching and vision therapy. Our research group published the results of a retrospective study demonstrating the benefit of a combined therapy of perceptual learning-based visual training and patching in children with moderate to severe amblyopia who did not recover vision with patching alone or had poor patching compliance [27]. We found a significant improvement in visual acuity and contrast sensitivity at one month after initiating treatment, with a stability of the outcomes during an 18-month follow-up. In our series, a stability of the outcomes achieved was also found during a two-year follow-up, confirming that this combined approach of treatment in amblyopia also promotes the absence of recurrences. Indeed, it should be considered that approximately one fourth of successfully treated amblyopic children with patching experience a recurrence within the first year of treatment [47]. Tang et al. [48] conducted a retrospective case series evaluating the results of patching for amblyopia management in Hong Kong, finding a recurrence rate of 7% and 46% in children with moderate and severe amblyopia, respectively.

The main limitation of the current study was its retrospective nature without the inclusion of a control group. Therefore, the results of the study must be considered with caution, and future randomized clinical trials should be conducted to confirm the outcomes presented here. Despite this limitation, to our knowledge, this is the series with the longest follow-up evaluating the results obtained with the combination of passive and active vision therapy in anisometropic and strabismic amblyopia. Furthermore, another strength of the study is that the same clinician performed all the evaluations as well as the active vision therapy sessions, minimizing the potential variability associated to the participation of different examiners. The use of a treatment protocol adapted to the peculiarities of each case can be considered as an additional limitation of the study, as the number of vision therapy sessions or hours of patching prescribed were not the same in all cases. However, we consider that the treatment in amblyopia must be customized according to the existing visual limitations, the presence of risk factors of a poor outcome, and the patient’s motivation and ability to follow the treatment plan. Furthermore, the compliance of the treatment can be considered as an additional limitation due to the impossibility of estimating it, as most home exercises were traditional exercises, such as flippers or printed sheets of tests, whose performance at home is impossible to be controlled. This may explain some variability among individuals in the time required to achieve a successful outcome. Finally, a BF score was used that was initially designed based on the Randot Preschool Stereoacuity Test (RPST) and the Randot Butterfly tests [17]. In contrast, due to technical limitations, the TNO test was used in our series. This may be considered as a limitation due to the difference of these tests with the TNO test, although the potential effect of this fact seems to be limited, considering that the first three pages of the TNO test are screening and provide a disparity of around 1900 arc seconds, and the Randot Butterfly test measures up to 2000 arcsec.

## 5. Conclusions

The combination of patching and active vision therapy is an efficacious approach for achieving an improvement in visual acuity and binocular function in anisometropic and strabismic amblyopia. However, the recovery achieved seems to be faster in anisometropic amblyopia, with a slower recovery of the binocular function in strabismic amblyopia if bifoveal fixation is ensured. Similarly, the results obtained were maintained during a two-year follow-up without recurrences. These results should be confirmed in future controlled clinical trials.

## Figures and Tables

**Table 1:** Statistical power calculations associated with the analysis of changes in distance BCVA and BF for the strabismic and anisometropic groups. Abbreviations: α, Type I error probability (probability that we will falsely reject the null hypothesis; δ, difference between means; σ, standard deviation of difference in the response of matched pairs; *n*, number of subjects; SP, statistical power. BCVA, best corrected visual acuity; BF, binocular function score.

	α	δ	σ	*n*	SP
*Strabismic amblyopia*					
Distance BCVA	0.05	−0.21	0.16	14	99.2%
BF	0.05	−1.27	1.20	14	95.2%
*Anisometropic amblyopia*					
Distance BCVA	0.05	−0.26	0.16	13	99.8%
BF	0.05	−0.67	0.56	13	97.3%

**Table 2:** Median [range], mean ± SD and (min-max) of the different variables evaluated in non-amblyopic (0) and amblyopic (1) eyes in the strabismic amblyopia group from the previous visit to 15 days, 30 days, 60 days and 90 days of treatment. Similarly, data of the one and two-year follow-up after completion of treatment were also added.

Median [IQ]	Previous		15 Days		1 Month		2 Months		3 Months		1 Year Post Avt		2 Years Post Avt			
Mean ± SD																
(Range)	0	1	0	1	0	1	0	1	0	1	0	1	0	1	*p* (0)	*p* (1)
Distance BCVA	0.00 [0.03]	0.19 [0.30]	0.00 [0.02]	0.11 [0.19]	0.00 [0.01]	0.07 [0.09]	0.00 [0.02]	0.03 [0.06]	0.00 [0.01]	0.02 [0.08]	0.00 [0.02]	0.02 [0.03]	0.00 [0.02]	0.02 [0.05]	0.73	<0.001
(logMAR)	0.03 ± 0.06	0.25 ± 0.17	−0.01 ± 0.09	0.17 ± 0.15	−0.00 ± 0.11	0.08 ± 0.06	0.01 ± 0.01	0.05 ± 0.05	0.01 ± 0.05	0.05 ± 0.07	0.01 ± 0.02	0.03 ± 0.03	0.00 ± 0.03	0.04 ± 0.05		
	(0.00–0.15)	(0.05–0.52)	(−0.30–0.10)	(0.00–0.52)	(−0.30–0.22)	(0.00–0.19)	(0.00–0.05)	(0.00–0.15)	(−0.08–0.15)	(0.00–0.22)	(0.00–0.07)	(0.00–0.10)	(−0.08–0.05)	(0.00–0.19)		
Near BCVA	0.00 [0.10]	0.20 [0.23]	0.00 [0.10]	0.15 [0.20]	0.00 [0.02]	0.10 [0.20]	0.00 [0.10]	0.10 [0.12]	0.00 [0.10]	0.00 [0.12]	0.00 [0.02]	0.00 [0.10]	0.00 [0.00]	0.00 [0.10]	0.11	<0.001
(logMAR)	0.03 ± 0.11	0.26 ± 0.20	0.03 ± 0.09	0.16 ± 0.13	0.01 ± 0.08	0.10 ± 0.10	0.01 ± 0.06	0.08 ± 0.10	−0.01 ± 0.06	0.03 ± 0.10	−0.01 ± 0.05	0.02 ± 0.06	0.01 ± 0.03	0.06 ± 0.08		
	(0.30–0.40)	(0.00–0.70)	(−0.10–0.20)	(−0.10–0.30)	(−0.10–0.20)	(0.00–0.30)	(−0.10–0.10)	(−0.10–0.30)	(−0.10–0.10)	(−0.10–0.20)	(−0.10–0.10)	(−0.10–0.10)	(0.00–0.10)	(0.00–0.30)		
Cycloplegic refraction (D)	3.00 [2.25]	4.94 [2.88]	3.00 [2.25]	4.94 [2.88]	3.00 [2.25]	4.94 [2.88]	3.00 [2.25]	4.94 [2.63]	2.94 [2.67]	4.75 [3.00]	2.94 [3.19]	5.13 [3.28]	2.88 [4.00]	5.00 [2.88]	0.74	0.93
	3.23 ± 1.83	4.12 ± 2.63	3.23 ± 1.83	4.12 ± 2.63	3.25 ± 1.86	4.08 ± 2.71	3.21 ± 1.87	4.10 ± 2.68	3.26 ± 1.90	4.11 ± 2.70	3.18 ± 1.94	4.13 ± 2.73	3.23 ± 2.18	4.11 ± 3.05		
	(0.50–6.63)	(−2.38–7.38)	(0.50–6.63)	(−2.38–7.38)	(0.25–6.63)	(−2.38–7.38)	(0.25–6.63)	(−2.25–7.38)	(0.50–6.63)	(−2.25–7.38)	(0.50–6.88)	(−2.25–7.63)	(0.38–6.75)	(−3.38–7.50)		
Subjective refraction	2.37 [3.06]	4.31 [3.38]	2.86 [3.06]	4.31 [3.72]	2.75 [3.06]	4.25 [3.06]	2.75 [2.78]	4.31 [3.16]	2.50 [3.44]	4.38 [3.41]	2.63 [3.44]	4.63 [3.38]	2.50 [3.75]	4.50 [3.19]	0.02	0.93
(D)	2.64 ± 2.13	3.55 ± 2.96	2.71 ± 2.13	3.60 ± 3.00	2.80 ± 2.04	3.55 ± 2.81	2.72 ± 1.97	3.61 ± 2.66	3.02 ± 2.00	3.72 ± 2.68	3.04 ± 2.04	3.82 ± 2.69	3.13 ± 2.38	3.74 ± 3.06		
	(−0.38–6.63)	(−3.00–7.50)	(−0.38–6.63)	(−3.00–7.50)	(0.00–6.63)	(−3.00–7.50)	(−0.13–6.63)	(−2.63–7.00)	(0.00–6.63)	(−2.38–7.00)	(0.00–6.50)	(−2.38–7.25)	(−0.50–6.75)	(−3.25–7.25)		
Distance Cover Test	3.00 [10]		3.00 [10]		2.25 [10]		3.75 [11]		2.25 [8]		0.00 [6]		0.00 [7]		<0.001	
(prism diopters)	5.14 ± 10.21		5.14 ± 10.21		3.89 ± 8.40		4.00 ± 10.13		2.18 ± 7.49		1.29 ± 7.09		2.64 ± 4.92			
	(−17–25)		(−17–25)		(−17–16)		(−25–16)		(−20–10)		(−20–10)		(−8–10)			
Near Cover Test	7.75 [10]		7.75 [11]		7.50 [11]		6.50 [8]		4.75 [9]		4.25 [9]		6.75 [10]		0.45	
(prism diopters)	7.50 ± 11.47		8.50 ± 11.67		6.32 ± 10.27		5.75 ± 9.94		3.99 ± 7.88		4.07 ± 8.46		6.89 ± 6.82			
	(−25–25)		(−25–25)		(−25–16)		(−25–16)		(−19–15)		(−19–19)		(−8–18)			
BF	4.00 [2.47]		4.00 [2.73]		4.00 [2.50]		2.60 [1.70]		2.60 [0.68]		2.30 [0.75]		2.30 [0.33]		0.002	
	3.79 ± 1.30		3.76 ± 1.33		3.62 ± 1.29		2.96 ± 1.09		2.29 ± 1.08		2.54 ± 0.75		2.52 ± 0.77			
	(1.60–5.00)		(1.60–5.00)		(1.60–5.00)		(1.60–5.00)		(1.51–5.00)		(1.80–5.00)		(1.70–5.00)			

**Table 3:** Median [range], mean ± SD and (min-max) of the different variables evaluated in non-amblyopic (0) and amblyopic (1) eyes in the anisometropic amblyopia group from the previous visit to 15 days, 30 days, 60 days and 90 days of treatment. Similarly, data of the one and two-year follow-up after completion of treatment were also added.

Median [IQ]	Previous		15 Days		1 Month		2 Months		3 Months		1 Year Post Avt		2 Years Post Avt			
Mean ± SD																
(Range)	0	1	0	1	0	1	0	1	0	1	0	1	0	1	*p* (0)	*p* (1)
Distance BCVA	0.00 [0.02]	0.22 [0.32]	0.00 [0.00]	0.07 [0.13]	0.00 [0.00]	0.02 [0.05]	0.00 [0.00]	0.02 [0.01]	0.00 [0.00]	0.01 [0.02]	0.00 [0.00]	0.01 [0.02]	0.00 [0.04]	0.00 [0.01]	0.2	<0.001
(logMAR)	0.01 ± 0.06	0.26 ± 0.17	0.01 ± 0.03	0.12 ± 0.10	−0.00 ± 0.02	0.04 ± 0.03	−0.01 ± 0.02	0.05 ± 0.08	−0.01 ± 0.02	0.03 ± 0.06	0.00 ± 0.01	0.02 ± 0.02	−0.02 ± 0.04	−0.00 ± 0.02		
	(−0.08–0.15)	(0.10–0.60)	(0.00–0.10)	(0.00–0.35)	(−0.08–0.02)	(0.02–0.10)	(−0.08–0.00)	(0.00–0.30)	(−0.08–0.00)	(0.00–0.22)	(0.00–0.02)	(0.00–0.07)	(−0.08–0.02)	(−0.08–0.02)		
Near BCVA	0.00 [0.10]	0.20 [0.20]	0.00 [0.05]	0.10 [0.25]	0.00 [0.00]	0.00 [0.10]	0.00 [0.05]	0.00 [0.15]	−0.10 [0.10]	0.00 [0.10]	0.00 [0.10]	0.00 [0.10]	−0.10 [0.10]	0.00 [0.10]	0.01	<0.001
(logMAR)	−0.01 ± 0.08	0.16 ± 0.18	−0.01 ± 0.05	0.06 ± 0.12	0.00 ± 0.00	0.04 ± 0.06	−0.02 ± 0.04	0.01 ± 0.09	−0.05 ± 0.05	−0.04 ± 0.06	−0.04 ± 0.05	−0.03 ± 0.05	−0.05 ± 0.05	−0.05 ± 0.05		
	(−0.10–0.20)	(−0.10–0.60)	(−0.10–0.10)	(−0.10–0.20)	(0.00–0.00)	(0.00–0.20)	(−0.10–0.00)	(−0.10–0.20)	(−0.10–0.00)	(−0.10–0.10)	(−0.10–0.00)	(−0.10–0.00)	(−0.10–0.00)	(−0.10–0.00)		
Cyploplegic refraction	1.88 [1.56]	4.38 [7.00]	2.00 [1.56]	4.38 [6.81]	1.88 [1.56]	4.38 [6.81]	1.88 [1.94]	4.38 [7.00]	2.00 [2.13]	3.25 [6.75]	2.00 [2.31]	3.63 [7.75]	2.00 [2.75]	3.38 [8.88]	0.59	0.31
(D)	2.21 ± 1.60	2.71 ± 4.10	2.22 ± 1.60	2.74 ± 4.06	2.19 ± 1.56	2.70 ± 4.00	2.31 ± 1.71	2.75 ± 4.02	2.27 ± 1.73	2.50 ± 3.92	2.11 ± 1.70	2.32 ± 4.53	1.89 ± 1.88	2.13 ± 4.58		
	(−0.50–6.25)	(−4.25–8.00)	(−0.50–6.25)	(−4.25–8.00)	(0.00–6.25	(−4.25–7.88)	(0.00–6.25)	(−4.25–7.88)	(0.00–6.25)	(−4.25–7.88)	(−0.50–5.50)	(−5.75–7.63)	(5.50–6.50)	(−6.00–7.13)		
Subjective refraction	1.25 [1.13]	3.50 [6.25]	1.00 [1.00]	2.50 [6.38]	1.13 [1.00]	4.00 [6.38]	1.13 [1.25]	3.00 [6.25]	1.25 [1.50]	3.75 [6.19]	1.50 [2.00]	2.63 [6.81]	1.38 [2.00]	2.38 [7.31]	0.9	<0.001
(D)	1.44 ± 1.48	2.29 ± 3.86	1.45 ± 1.47	2.13 ± 3.89	1.45 ± 1.44	2.22 ± 3.94	1.55 ± 1.49	2.06 ± 3.94	1.54 ± 1.51	2.11 ± 3.99	1.62 ± 1.49	1.90 ± 4.11	1.51 ± 1.61	1.49 ± 4.17		
	(−0.25–5.50)	(−4.38–7.50)	(−0.25–5.50)	(−4.50–7.50)	(−0.25–5.50)	(−4.50–7.38)	(−0.25–5.50)	(−5.00–7.38)	(−0.25–5.50)	(−5.13–7.38)	(−0.25–5.25)	(−5.38–7.25)	(−1.00–5.00)	(−5.50–7.00)		
Distance Cover Test	0.00 [0]		0.00 [0]		0.00 [0]		0.00 [0]		0.00 [0]		0.00 [0]		0.00 [0]		0.04	
(prism diopters)	−0.69 ± 1.80		−0.62 ± 1.56		−0.86 ± 0.83		0.23 ± 0.83		−0.38 ± 1.39		−0.38 ± 1.39		0.00 ± 0.00			
	(−6–0)		(−5–0)		(0–3)		(0–3)		(−5–0)		(−5–0)		(0–0)			
Near Cover Test	0.00 [2.8]		0.00 [3.3]		0.00 [0]		0.00 [0]		0.00 [5]		−4.00 [4]		0.00 [2]		0.04	
(prism diopters)	0.15 ± 3.78		0.23 ± 3.85		−0.62 ± 3.50		−0.62 ± 3.50		−1.69 ± 3.64		−2.62 ± 2.76		−1.23 ± 2.52			
	(−10–4.5)		(−10–4.5)		(−10–6)		(−10–6)		(−8–4)		(−8–0)		(−8–0)			
BF	2.30 [0.50]		2.10 [0.66]		2.00 [0.69]		2.00 [0.44]		2.08 [0.59]		1.80 [0.14]		1.80 [0.00]		<0.001	
	2.52 ± 0.69		2.43 ± 0.75		2.19 ± 0.64		2.01 ± 0.34		2.06 ± 0.33		1.89 ± 0.33		1.85 ± 0.26			
	(1.80–4.00)		(1.70–4.00)		(1.60–4.00)		(1.40–2.70)		(1.60–2.70)		(1.51–2.70)		(1.70–2.70)			

## Data Availability

Not applicable.
